# Mothers' experience of infant massage in child health care: A qualitative interview study

**DOI:** 10.1002/nop2.2206

**Published:** 2024-06-14

**Authors:** Marlene Danielsson, Hanna Hansson Lustig, Pernilla Garmy, Eva‐Lena Einberg

**Affiliations:** ^1^ Department of Nursing and Health Sciences, Faculty of Health Sciences Kristianstad University Kristianstad Sweden; ^2^ Department of Health Sciences, Faculty of Medicine Lund University Lund Sweden

**Keywords:** child health care, infant massage, interviews, mothers, qualitative study

## Abstract

**Aim:**

To investigate mothers' experience of infant massage.

**Design:**

This was an exploratory‐descriptive qualitative study based on individual interviews.

**Methods:**

A qualitative interview study with an inductive approach was used according to the COREQ guidelines. The participants in the study were mothers (*n* = 11) residing in Sweden who received training in infant massage from the child health care nurse in the child health care services. The transcribed interviews were analysed using a qualitative content analysis.

**Results:**

The collected material resulted in two categories and eight subcategories. The categories were *learning infant massage* and *using infant massage as a tool*. The eight subcategories were massaging in a parent group, massaging at home, massage movements and the child health care nurse's supporting hand, reading the child's signals, creating time and relaxation together, interaction and connection between the child and the parent, relief from stomach problems and anxiety and continuing to massage the older child.

The study showed that mothers experienced that the relationship created through infant massage brought more joy, tenderness and security to the child. The child health care nurse had an important role in supporting the mothers, especially when it came to different views on doing infant massage at home and in groups.

**Patient or Public Contribution:**

Mothers with experience of infant massage were interviewed.

## 1 INTRODUCTION

Touch is the first sense that develops in humans (Di Plinio et al., 2022) and how the infant can sense the parents' mood through touch (Cheng et al., 2011). Touch releases the hormone oxytocin, which can have a positive effect on the attachment between child and parent (Moberg, 2019). Infant massage is structured strokes over the infant's body (McClure, 2017), and at some Child Health Services (CHS), the CHS nurse teaches the parents massaging their infant in various movements from the feet to the face and back (Sylvie, 2015).

A systematic literature review by Mrljak et al. (2022) compiles research from six different countries between 2017 and 2021 showing that infant massage has a positive effect on pain, including postoperative pain and colic pain, and can positively affect bilirubin among infants who have jaundice. The infant massage was also seen to have some effect on weight gain in infants (Mrljak et al., 2022).

Infant massage has been shown to have a positive impact on both mothers' mental health and the bond between mother and child (Shoghi et al., 2018). Mothers with postpartum depression who perform infant massage feel better at an earlier stage (Afand et al., 2017). McCarty et al. (2023) found in a systematic review that maternally administered infant massage may benefit mothers of preterm infants by reducing anxiety, stress and depressive symptoms, and by improving maternal–infant interactions. Regular infant massage could strengthen the bond between child and parent and give mothers a break from everyday life to spend time with the baby (Midtsund et al., 2019).

There are many challenges with becoming a parent and postpartum depression can affect the bond between parent and child. According to Bowlby's (1979) attachment theory, children who have had parents who were predictable, responsive and warm in early contact become secure in themselves. Johansson (2019) shows that postpartum depression occurs in both mother and father; however, postpartum depression can occur at different stages after the child is born. Postpartum depression can cause the parent to spend less time with their child and thus affect the bond between child and parent (Field, 2010). The ability to create security and provide care is crucial to contributing to the child's good upbringing. Conditions differ between the mother and the father when it comes to creating a close relationship with their baby, and infant massage can help the father bond with his baby (Cheng et al., 2011). The child health care nurse has a crucial role to offer various supports to parents to help them form a close relationship with their infant, for example, by teaching the parents to practise infant massage.

The main task of child health services (CHS) is to monitor and promote the health status, life situation and development of all children (National Board of Health and Welfare, 2014). It is important to follow up children within particularly vulnerable groups to create equal health. The National Board of Health and Welfare (2014) emphasizes the importance of strengthening children's rights according to the Convention on the Rights of the Child. CHS includes child health care centres, family centres, social services and open preschool for children aged 0–6, while school health takes over when the children start school (Wettergren et al., 2016). Child health care services are staffed by Registered Nurses with specialization in public health and children and young people, also known as child health care nurses. Their main task is to promote children's health and prevent ill health (Lefevre, 2016).

The child health care nurses' mission also includes supporting the parents in their parental role through parenting support to promote the child's health and psychosocial development (Lefevre, 2016). Parenting support can be provided individually or in groups, and it is important that the child health care nurse is sensitive to what suits the individual parent (Swedish Association of Local Authorities and Regions, 2019). Part of the parenting support is an invitation to a parenting group where parents can meet other parents and share experiences. However, it can be difficult to reach the parents who are most in need of parental support, especially those who come from other countries and cultures (Skoog, 2022). The child health care services in Sweden reach almost 100% of families regarding individual health visits and checkups however, only about 50% of those invited to attend parental groups (Lefevre, 2016).

Infant massage can be offered as part of the parenting support in child health care services (Swedish Association of Local Authorities and Regions, 2019) but it is important that the parents feel comfortable with it and are not stressed during the performance (Isaksson et al., 2023). The parents' participation in the early care of the child can contribute to a higher tendency to get involved with their child when he gets older (Bowlby, 1979).

According to the Swedish National Handbook for CHS (Swedish Association of Local Authorities and Regions, 2019), it is important that parents get involved in the infant's care to increase their participation and commitment during the child's upbringing. Infant massage can be used as a tool to achieve this. Further qualitative research within the Swedish context is required to understand parents' experiences of infant massage.

## 2 AIM

To investigate mothers' experience of infant massage.

## 3 METHODS

A qualitative interview study with an inductive approach to investigate mothers' experiences of infant massage was used. The study was conducted according to the COREQ guidelines (Tong et al., 2007).

Ethical considerations for qualitative research guided the current research design and practice. These principles included anonymity, voluntary participation, privacy and confidentiality (Sanjari et al., 2014). The study was approved by the Swedish Ethical Review authority before data collection started. All methods were carried out in accordance with the human rights principles outlined in the Declaration of Helsinki (WMA, 2013). Written informed consent was obtained from all participants.

### 3.1 Setting

The participants in the study were mothers residing in the southern part of Sweden who received training in infant massage from child health care services either regionally or privately owned. The training was provided by child health care nurses who were trained by the International Association of Infant Massage (IAIM).

### 3.2 Sample

Purposeful sampling was used to obtain data. Inclusion criteria were parents over the age of 18 who received training in infant massage via CHS in southern Sweden when their babies were 2–6 months old. Initially, information about the study was sent to child health care services with infant massage in five municipalities. When there was a low response, the area was expanded and more information was distributed on social media, open preschools and family centres. Eleven interviews were finally conducted (see Table 1). Although both mothers and fathers were invited to the study, only mothers participated. The informants were women aged 29–40 (mean age 33.6 years), born in Sweden or abroad. All of the participants had studied at university level.

**TABLE 1:** Information about the participants.

Interview No	Sex	Age	Age of the child at the start of the infant massage course
1	F	32	3 months
2	F	32	6 months
3	F	29	2.5 months
4	F	30	6 months
5	F	37	3 months
6	F	40	2 months
7	F	33	6 months
8	F	33	3 months
9	F	37	3 months
10	F	34	2 months
11	F	33	4 months

### 3.3 Data collection

A semi‐structured interview guide was used (see Appendix). Examples of questions were “Can you tell us about your experiences with infant massage?” and “Can you tell us about the benefits/disadvantages of infant massage?” A pilot interview was conducted to test the technical equipment and interview setup; however, this interview was adequate and was included in the analysis. The informants received a consent form and the interview guide in advance. Written informed consent was obtained before data collection started. The interviews were conducted via the digital tool Zoom and saved on a USB stick. The interviews were 20–40 min and were conducted by the first and second authors between September 2022 and January 2023. The interviewers were both female Master's level researchers with several years of experience as Registered Nurses. All interviews were transcribed verbatim by the first and second authors.

### 3.4 Analysis

The results from the present study have been analysed using a qualitative content analysis that follows Graneheim and Lundman's (2004) interpretation. According to Graneheim and Lundman (2004), the transcribed interviews were the analysed material. The first and second authors transcribed the interviews separately and then read the text together and analysed the results by picking out meaning units. Graneheim and Lundman (2004) describe a meaning unit as the part of the text that is considered supportive and informative for the purpose of the study. After the authors had taken out meaning units that answered the purpose of the study, these were processed and condensed into a shorter text where the central part of the text emerged. The condensed text was converted into codes that served as a label to describe the content (see example of the analysis in Table 2). To really obtain correct information from the interviews, the texts were processed several times jointly by the first and second authors. The first and second authors then cut out all the codes together with the condensed sentence units and sorted out by hand which codes fit together to create subcategories and later categories. The first and second authors discussed codes, subcategories and categories with the last author to move the analysis forward and ensure trustworthiness. Graneheim and Lundman (2004) describe that subcategories and categories are there to describe the content of the results. When the authors processed all the material, the categories emerged. The analysis was discussed by all four authors until a consensus was obtained.

**TABLE 2:** Example of the analysis process.

Extracts from the interviews	Condensed text	Subcategory	Category
*It felt like she (the child health care nurse) really explained how to do it. Uh… and there weren't any strange things like turning the baby or doing anything, but very simple light movements*	The child health care nurse explained that there were very simple light movements	Massage movements and the supporting hand of the child health care nurse	Learning infant massage
*But then we don't do it every night, it's not something I feel we have to do, it's more that yes now it would be appropriate, it's getting to be time but not really, he probably won't be able to fall asleep for the night yet, yes but then we try to relax here or…*	He probably won't be able to fall asleep but then we try to relax here with infant massage	Relief from stomach problems and anxiety	Using the infant massage as a tool

## 4 RESULTS

The collected material resulted in two categories and eight subcategories (see Table 3). The categories were *learning infant massage* and *using infant massage as a tool*. The eight subcategories were massaging in a parent group, massaging at home, massage movements and the child health care nurse's supporting hand, reading the child's signals, creating time and relaxation together, interaction and connection between the child and the parent, relief from stomach problems and anxiety and continuing to massage the older child.

**TABLE 3:** Categories and subcategories identified in the results.

Categories	Subcategories
Learning infant massage	Massaging in parent group
	Massaging at home
	Massage movements and the child health care nurse's supporting hand
	Reading the child's signals
Using infant massage as a tool	Creating time and relaxation together
	Interaction and connection between the child and the parent
	Relief from stomach problems and anxiety
	Continuing to massage the older child

### 4.1 Learning infant massage

The mothers described that infant massage could be offered via parenting groups at child health care services where approximately six to eight parents with children participated. According to the mothers, the majority of participants in the parent group were first‐time mothers. The course lasted between 1 and 3 months. Although there were challenges such as carrying out the massage in a group with other parents or making time for it at home, infant massage was experienced as something positive. Initially, the mothers worried about doing it right and not harming the child; however, information and support from the child health care nurse alleviated this. The mothers became flexible and sensitive to their child's needs.

#### 4.1.1 Massage in parent group

The mothers described a calm and atmospheric atmosphere at the massage sessions at child health care services, which they felt the children enjoyed. Two mothers who had taken the infant massage course with their partners found it a positive experience for both them and their partners. A mother described how the experience of massaging in a group gave her a positive feeling and it was a nice opportunity to see the children lying in a circle and interacting and developing together during the massage sessions. Another mother felt that it was good that the room was heated during the massage as the children would be undressed. The first massage session at the child health centre was described as positive by one mother:

… I remember she [the child] became very like this like “what are you doing? What's going on?” and then you could really see how she thought like “what is this but it's quite okay… well or wait now” or something like that… ah she was so cute. So it was positive right from the start. (#10#)

One mother described that it was nervous and tense the first time the massage was to be performed in the parent group, but that it became more relaxed over time. Another mother stated that there was much that could feel stressful during the massage session, for example, that the child could urinate, which made the parent feel that it was difficult to relax. There were mothers who experienced difficulties in being able to relax and take care of a child who was screaming, while other mothers were more comfortable in the situation. One mother described that there were several moments of disturbance when the infant massage was performed in a group. Her child wanted to look at all the other children and it was difficult to focus, despite this she still had a positive experience from the massage. Another mother said that there were several mothers from different cultures in her parenting group and that she found it difficult to get into the group. Several mothers expressed that it is difficult to make time to attend the infant massage course at child health care when they had several children, the logistics and practicality did not always work. Some mothers pointed out that it could be difficult to have time to fit when you have an infant, everything must be in order, the child must be satisfied, have eaten and have a new nappy.

Many mothers expressed their gratitude for being offered the infant massage as part of the parenting group at the child health care service. Some of the mothers described that they had chosen to take the infant massage course because they found it interesting to learn new things and some thought it was a good opportunity to spend more time with their baby. Other mothers saw the infant massage as a good opportunity to meet other parents. A mother describes the invitation to the infant massage course as follows:

…I don't think I had, it's not certain that I would have looked it up on my own, but now that I was served it, I felt that it really became a wow feeling. (#3#)

A mother who only attended infant massage on one occasion learned infant massage for 2 h and felt she gained enough knowledge to use it extensively with her children at home. Another mother who attended infant massage with both her children describes how she experienced the difference between the different sessions, with the eldest child it was only one session of infant massage while for the second child was a course with a trained instructor in infant massage. She felt that to some extent it ran into the sand on the first occasion and that she gained deeper knowledge on the second occasion. She describes the experiences of the various designs as follows:

… it was probably the first time there that we didn't get to learn it for real or something, so it was more just… it felt so half‐hearted in some way to only learn it like that in quick circumstances if you say so, it was only on one occasion for like an hour or whatever it was that we met like… so if you had learned it more in depth then, we would certainly have been able to use it in a different way with the first child as well. (#6#)

#### 4.1.2 Massage at home

There were several mothers who described how they performed the infant massage at the changing table at home as they considered it easy because the baby was already undressed; however, it was difficult to massage if it was too cold inside. A mother expressed that she took the opportunity to check the child's whole body when she was massaging, she felt it was a good way to do a small inspection of the child. Several mothers felt that it was difficult to get a quiet moment for the infant massage as there were many distractions at home. It was also felt to be difficult to get the massage in as a routine due to, among other things, the siblings in the family. According to one mother, the massage did not happen as often as she would have liked. However, another mother said that the infant massage could be done anywhere and anytime as she always had her baby and her hands with her. It also emerged from several mothers that they had shown their partner's infant massage at home and that some partners then applied it, while some had no interest in learning infant massage. One mother said that both she and her partner used the infant massage at times, when there was time. Another mother describes it this way:

And then there's probably that everyday puzzle, taking the time because it's not, it's not going to be good if it's something you change because you have to and then it should go away quickly, because you need the time. (#11#)

#### 4.1.3 Massage movements and the supporting hand of the child health care nurse

Fear and discomfort to perform certain movements were something that some mothers mentioned, while the majority of mothers described that the children were not as fragile as first thought. Several mothers said that they had received written information about the movements as support when massaging at home, this made several feel more comfortable massaging. Some mothers described that they felt worried about the movements at the beginning, but that they received good support from the child health care nurse who allowed them to relax. Another mother described that she wanted to do the massage right from the start, but had to end up being able to try things out, she felt that the important thing was that the child was close. The majority of mothers expressed that not all massage movements suited their children. It could be a challenge to remember all the movements according to one mother, while another mother did not find the movements complicated. One mother described her experience of the movements in the infant massage as follows:

It felt like she (the child health care nurse) really explained how to do it. Uh… and there weren't any strange things like turning the baby or doing anything, but very simple light movements. (#9#)

#### 4.1.4 Reading the child's signals

Mothers expressed that they asked their children for permission to massage before they started, in this way, the child was prepared for the massage. One mother felt it was important to accept that the baby was not always receptive to infant massage. Another mother pointed out that the massage always took place on the child's terms and that as a mother you learned to read the child's signals whether the movements were appreciated or not. The mother expressed that the massage could be very intimate after all. Another mother said that she trusted herself, and that she could tell when the child did not want to be massaged anymore. This is how one mother described how she read her child's signals:

Uh… and you kind of noticed that he was lying and smiling, showing that I like this. Above all, he became very calm and lay still. And then when he showed that he didn't want to, he started moving and grumbling and whining and really no, now I don't want this. (#2#)

One mother found that her child was quite impatient and did not want to be massaged for long before he got tired. Furthermore, a mother expressed that her child had difficulty accepting lying on his stomach, but if she first massaged his stomach and then his back, he accepted lying on his stomach in a different way. Another mother says that sometimes it worked well to massage harder depending on how the child reacted. One mother described that the first child clearly showed no need to be petted or carried and did not enjoy the infant massage, while the second child enjoyed all forms of touch and so did the massage. One mother described it this way:

…above all, babies also have different preferences, so not everyone likes it (infant massage), it's not as easy to massage my daughter as it is to massage her daughter, it can be very different, not everyone likes it on the same way and some children are somehow more difficult to make that contact with… (#11#)

### 4.2 Using the infant massage as a tool

The mothers described infant massage as a cosy moment that also may be a useful tool. Infant massage is something pleasant beyond everyday necessities. It gives an opportunity for the child and the mother to be together and create interaction and connection, as well as relaxation for both of them. Furthermore, according to the mothers, infant massage can be used as a tool to give the child relief from stomach problems and anxiety. Mothers described that they continue to massage the older child, even the child themselves may request the massage.

#### 4.2.1 Creates time and relaxation together

Several of the mothers expressed that the infant massage created a cosy moment of private time between the child and the mother. One mother told how she and her children could start the day with a morning bath and then a massage session. According to the mother, this created a relaxed feeling, which she carried with her throughout the day. Another mother said that the massage was not a requirement but that it was nice when it was done, it was a bonus; however, nothing negative was experienced if they did not have time for the massage. Other mothers also described that the massage was something cosy that was carried out in addition to all the other musts such as breastfeeding and nappy changes. A mother told how she could use the infant massage as a tool to be able to spend time with her child, but also that it was a way to relax herself. The mother continued and described the time spent with their child through the infant massage as follows:

I thought it could provide a kind of cozy moment, with the baby as well, and a present moment above all that you felt presence with your… with your child. Oh, and I also thought it was nice to have a tool that you still had with you… all the time. You always had your hands… your hands and the child with you wherever you were, so you could still apply it everywhere. (#7#)

A mother expressed that in situations when the child was inconsolable, it was easy to feel helpless and that the infant massage in these situations created a meditative feeling that made the mother calm, this calm was felt to be transferred to the child. Several other mothers also experienced this and expressed that the infant massage created a focus on the child. One mother described that it was like being in a “bubble” when she massaged her child, the child could focus on her without being distracted by her surroundings. The child was perceived to seek eye contact with his mother when receiving the massage according to the mother, which created a sense of calm for the child and mother. One mother described the calm like this;

…and after this all babies probably have periods when they scream a lot and it (infant massage) kind of calms both parent and baby and I think it's great. (#11#)

Several mothers described that they became calm and harmonious by massaging a small baby, it created relaxation for both the child and the mother, and some mothers told that their child could fall asleep from the massage. The infant massage was experienced by several mothers as something positive for both them and their children as they felt that they could take advantage of the time with their child. One mother shared that finding time for her second child could be difficult, but that infant massage allowed her to focus on her baby for a while.

#### 4.2.2 Interaction and connection between the child and the mother

The majority of mothers said that they felt that the infant massage created a special connection between them and the baby, that it was a way to communicate and interact with their baby. Most of the mothers felt that they had been able to bond in a good way with their first child through the infant massage and thus chose to massage their next children as well. One mother said that although the massage did not take place for an extended period of time, she felt that she was able to bond with her child in that moment, she felt that it felt natural and that it strengthened their relationship. Another mother described what infant massage had done for her like this:

…because it feels like it (the infant massage) strengthens the relationship between the two of us, well I think absolutely and as I said, it's only me who does it so it becomes our thing in some way. Erm our own little thing… (#8#)

Several mothers said that the touch was a good way to create a relationship and closeness to their child, they also experienced the infant massage as an opportunity for both mothers to get in better contact with the child. Two mothers said that the fathers of their children also went to the infant massage with them, they said that the massage was experienced as an important part of the connection for the fathers. A father had long working days and when he came home the infant massage could be a moment for the father and the child to bond according to the mother's experience. Another mother described her experience of the father massaging like this:

yes, and also his father also massaged him and he also said that man, it was a very cozy moment together, it had a positive effect on the parent‐child relationship because it was somehow strengthened. (#2#)

One mother described that she placed a lot of value in how she experienced touch and being touched herself, and through that experience, she thought it was a matter of course to massage her child. One mother described that the father was not comfortable performing the infant massage and therefore did not use it. Another mother described that the infant massage had become something that the whole family was involved in, with the older siblings participating and massaging each other while the mother massaged the baby. The mother described it like this:

…it's a good thing to get closer to each other in one… even if they're a little older, so it doesn't have to be exactly a baby massage. Uh… plus it will be like a nice little moment together so it is… or a cozy moment together as well. Er… so it's probably more that we can sort of, well… it's just going to be a moment together. (#5#)

#### 4.2.3 Relief from stomach problems and anxiety

All mothers described that they had used the infant massage to relieve stomach aches and gas when the children had experienced it bothering them. Several mothers described that they felt that their children understood that the massage would help when they had a stomach ache and the mothers then felt that the children relaxed when the massage started. Some mothers expressed that their children became constipated when they started eating regular food, and the massage movements over the stomach helped with the constipation. One mother expressed that the infant massage was used more intensively on the days the child felt constipated, after that, the massage could be used less intensively to maintain the stomach. A mother told about the first occasion when the child was helped by the massage for constipation;

Yes, that would probably be the first time, the first time that uh, that I noticed it really helped with constipation. Uh and I heard that now, the stomach started working and he could poo. Er… when I actually got confirmation that wow, it works. (#2#)

Mothers also reported that the infant massage was used to calm the child in case of anxiety. A mother tells us that her child sometimes had tantrums that were difficult to break except through the massage, which immediately had an effect and the child became calm. Several mothers described how their children were also calmed down when the music from the massage sessions started to play as the mothers felt that they connected this to the massage. There were also mothers who described that their children could recognize the massage movements and the mothers felt that the children felt a sense of security through this, which resulted in a sense of calm. One mother told how she and her partner used the massage to calm the child when he was worried at night. One mother described how her child was calmed by the massage like this:

…and that she also became a little calmer because of it. So that now I mostly use the back massage in the evening… well, when laying down. So that she sort of… well, you notice that she relaxes and finds it easier to calm down. (#4#)

Several mothers expressed that their children were anxious when lying down and that the massage then created a quiet moment, it became a come‐to‐relax thing. Many mothers further described that they felt that the infant massage had worked well as an evening routine to alleviate the anxiety that they had, therefore, chosen to continue with the infant massage for a longer period. One mother described that when her child started to get whiny and tired in the evening, she could massage to break the anxiety and get the child to settle down, she further described it like this:

But then we don't do it every night, it's not something I feel we have to, it's more that yes now it would be appropriate, it's getting to be time but not really, he probably won't be able to fall asleep for the night yet, yes but then we try to relax here with infant massage… (#3#)

#### 4.2.4 Continue massaging the older child

Several mothers described that their children have continued to enjoy being massaged as they have gotten older. According to several mothers, the infant massage followed until the children stopped wearing diapers and then became a natural part of life; however, it is not done regularly. However, several mothers felt that it could be difficult to massage when the child got a little older, as it no longer lay still in the same way. The massage could also be experienced more like a game for the child according to some mothers. Another mother says that she will continue with the massage when her children are older because it gives them their own time together. One mother says her 10 years old still comes to her asking to be massaged. One mother described her son asking for the massage like this:

Just like big brother still likes getting a massage. Yes, he comes up and wants to be massaged and so on, and now he is two years and eight months old. (#1#)

## 5 DISCUSSION

The present study examined mothers' experience of infant massage. The discussion will focus on three main findings: massaging in a parent group, creating time and relaxation together and interaction and connection with the infant and parent. The mothers felt that it was positive to meet other parents and children during infant massage, but it was not always easy to massage their child in a group as the child could be disturbed by the surroundings. The infant massage created relaxation and cosiness between parents and children, and many mothers used the infant massage as a relaxing evening routine. The study also showed that the infant massage created time for parents to focus on their child and created a focus between parents and children.

The first finding discussed is the mothers' experiences of infant massage in a parenting group. Several mothers in the study thought it was positive to meet other parents and children during the massage sessions and that this could help create relationships between the parents. Lotfalipour et al. (2019) found that mothers who performed massage on their preterm infants showed greater improvement in their mood compared with those in the control group. Khuzaiyah et al. (2022) report similar results in their study of mothers' experience of an online infant massage course. However, some mothers in the present study felt stressed about massaging their children in a group due to interference from other children. Another study by Midtsund et al. (2019), who focused on mothers who felt stress about parenting, found similar results where mothers experienced stress about their child disturbing during the massage session. Another observation that Isaksson et al. (2023) did in their study was that several parents felt stressed that the parent group meetings were too long and that they did not have time to participate. The length of parent group meetings should be taken into account when planning. Furthermore, based on the present study, child health care nurses should facilitate parents' participation, that is, reduce their stress by addressing and normalizing that children can be disruptive and that it is allowed.

The other main finding discussed is that infant massage can create time and relaxation for both parents and their babies. An interview study conducted by Chan et al. (2018) show that mothers who perform infant massage experience that their children become more relaxed and sleep better with fewer awakenings. The parents in the present study also felt that the infant massage gave them time with their children and that they could use the massage as a relaxing evening routine. A similar study by Midtsund et al. (2019) also showed that infant massage can create time for parents and their children to be present together.

The infant massage can be performed anywhere and at any time, which can be an important argument for child health care nurses to recommend the massage to parents. This may be particularly important because Isaksson et al. (2023) study showed that some parents did not prioritize infant massage due to lack of time. Several studies show that infant massage can be a relaxing activity for both parents and their babies and that it can create time for present moments between parents and babies. Child health care nurses may, therefore, consider recommending infant massage to parents and emphasizing the benefits of the massage being performed anywhere and anytime.

The third main finding discussed is parents' experience of interaction and connection between children and parents through infant massage. The results showed that the infant massage helped create a stronger relationship and connection within the family. Previous studies by Chan et al. (2018) have also demonstrated similar results, where the infant massage strengthens the relationship within the family and functions as a valued family activity.

Touch through massage can be perceived in different ways, depending on how the individual values the touch. However, all parents in the study stated that the touch through the infant massage created a special contact and was a good way to interact and bond with their child. Parents in Midtsund et al. (2019) study reported that the infant massage helped to bond with their child, especially during periods of breastfeeding problems, colic or when the child was often sad. This is important because Bowlby (1979) points out that the attachment between parent and child plays a large role in the child's development of own relationships and attachment patterns in the future.

A child health care nurse has an important role in identifying difficulties in the attachment between child and parent and supporting parents in their parenting to promote attachment. According to the Swedish National Handbook for CHS (Swedish Association of Local Authorities and Regions, 2019), infant massage can be offered as part of parenting support to contribute to increased attachment.

A study by Cheng et al. (2011) showed that infant massage can reduce fathers' stress and increase attachment to their infants. The authors suggest that fathers can also benefit from various parenting educations to strengthen their attachment to their children.

The results of the present study are in line with what is recommended in the Swedish National Handbook for CHS (Swedish Association of Local Authorities and Regions, 2019) that child health care nurses should work to promote the relationship between children and parents through various methods, including infant massage.

### 5.1 Strengths and limitations

The study's strengths include a strategic selection of informants to obtain information‐rich data that answers the study's purpose. An appropriate data collection method was chosen to study people's experiences of various phenomena. A careful and methodical analysis process that included both manifest and latent analysis was carried out by the four authors to strengthen the reliability of the results. Weaknesses of the study include the inclusion criteria that limited the recruitment of informants to a specific area which could have affected the validity of the results. Fathers' experiences were not included in the study. All informants were women with a university degree which may have affected the generalizability of the results.

## 6 CONCLUSION

The study showed that mothers experienced that the relationship created through infant massage brought more joy, tenderness and security to the child. Infant massage was perceived by the mothers as positive for both child and parents, who used it to bond, interact and spend more time with their children. It also provided relaxation and relief from stomach problems and anxiety. The child health care nurse had an important role in supporting the parents, especially when it came to different views on doing infant massage at home and in groups. The study also showed that more research is needed regarding the fathers' experience of infant massage in a Swedish context. This would be beneficial to the child health care nurse's work in involving both parents in the child's care.

## 7 STATEMENT OF RELEVANCE TO THE FIELD

It is important that parents get involved in the infant's care to increase their participation and commitment during the child's upbringing. Infant massage can be used as a tool to achieve this. Further qualitative research within the Swedish context is required to understand parents' experiences of infant massage.

## AUTHOR CONTRIBUTIONS

Study design: PG, EE. Data collection: MD, HL. Analysis: MD, HL, PG, EE. Writing the first draft: MD, HL, PG. Revising manuscript: MD, HL, PG, EE.

## FUNDING INFORMATION

The study was supported by Gyllenstiernska Krapperup Foundation.

## CONFLICT OF INTEREST STATEMENT

There are no conflicts of interest to declare.

## ETHICS STATEMENT

The study was approved by the Swedish Ethical Review authority before data collection started (2022‐00610‐01). All methods were carried out in accordance with the human rights principles outlined in the Declaration of Helsinki. Written informed consent was obtained from all participants.

## CONSENT FOR PUBLICATION

NA.

## Data Availability

The data that support the findings of this study are available on request from the corresponding author. The data are not publicly available due to privacy or ethical restrictions.

## Semi‐structured interview guide

### 1

- How did you decide to learn infant massage?
- Can you tell us about your experiences with infant massage?
- What does your child think about receiving an infant massage?
- Does anything happen to your child when you give a massage?
- Can you tell us about the benefits of infant massage?
- Can you tell us about disadvantages of infant massage?
- Are there challenges to using infant massage?
- Is there anything else you would like to add?
